# 小剂量多西他赛持续化疗联合树突状细胞生物免疫治疗抑制VEGF分泌

**DOI:** 10.3779/j.issn.1009-3419.2013.09.04

**Published:** 2013-09-20

**Authors:** 严 周, 慧敏 王, 华 钟

**Affiliations:** 200030 上海，上海交通大学附属上海市胸科医院呼吸内科 Department of Pulmonary Disease, Shanghai Chest Hospital, Shanghai Jiaotong University, Shanghai 200030, China

**Keywords:** 小剂量持续化疗, 生物免疫治疗, 肺肿瘤, Metronomic chemotherapy, Dendritic cell vaccine, Lung neoplasms

## Abstract

**背景与目的:**

肺癌生存期有限，需要探索一种新颖的治疗模式。本研究应用免疫治疗联合小剂量化疗的方法，观察联合治疗的抑瘤效应。

**方法:**

C57/BL6J小鼠负荷3LL肿瘤，按照不同的治疗模式：对照组、小剂量持续化疗组、树突状细胞（dendritic cells, DCs）生物免疫治疗组和小剂量化疗联合DC生物免疫治疗组；观察不同治疗模式下小鼠的生存时间；应用微渗析分析联合液相芯片分析瘤体内bFGF和VEGF的分泌。

**结果:**

接受小剂量化疗联合DC生物免疫治疗组的小鼠中位生存时间为（27.6±3.2）天，与对照组（13.5±2.7）天、生物免疫治疗组（13.1±2.3）天、小剂量持续化疗组（11.8±3.0）天间比较，差异具有统计学意义（*P* < 0.05）。联合治疗组瘤体内在48 h、72 h内VEGF的分泌下降，较其余治疗组的差异具有统计学意义（*P* < 0.05）。

**结论:**

小剂量化疗联合DC生物免疫治疗能抑制肿瘤内VEGF的分泌。

## Abstract (en)

**Background and objective:**

The survival rate of lung cancer is low, thus new methods for treating this form of cancer must be explored. This study applies immune therapy with metronomic chemotherapy to observe the effect of combined therapy on suppressing tumor.

**Methods:**

Mice were inoculated with Lewis lung carcinoma cells. Different treatments, namely, saline, metronomic chemotherapy, dendritic cell (DC) vaccine, and metronomic chemotherapy with DC vaccine were administered to corresponding mice groups. Basic fibroblast growth factor and vascular endothelial growth factor (VEGF) were detected via microdialysis and Luminex.

**Results:**

The median survival time of mice in the metronomic chemotherapy with DC vaccine group was (27.6±3.2) days, whereas that in the saline group was (13.5±2.7) days (*P*=0.008), that in the DC) vaccine group was (13.1±2.3) days (*P*=0.01), and that in the metronomic chemotherapy group was (11.8±3.0) days (*P*=0.01). The mice in the metronomic chemotherapy with DC vaccine group exhibited longer survival time than the mice in the other groups. This result could be related to the downregulation of VEGF secretion in the tumor-bearing mice groups within 48 h to 72 h.

**Conclusion:**

VEGF secretion could be downregulated in tumor-bearing mice by administering metronomic chemotherapy with DC vaccine.

肺癌是我国最多发的一种恶性肿瘤，极易发生侵袭和远处转移，约75%的患者就诊时已处于晚期阶段（Ⅲ期或Ⅳ期），5年生存率仅为30%左右^[1, 2]^，死亡率居恶性肿瘤死亡率首位。肺癌的远处转移是造成其生存率低下的主要原因之一。因此，深入探讨肺癌的侵袭和转移机制，寻求有效抑制侵袭转移的方法，是当前焏待解决的问题。迄今针对肿瘤瘤体本身的传统多学科综合治疗，如手术、放疗、化疗等，被证实无法突破决定肺癌患者生存的颈瓶—癌细胞转移，我们需要重新审视传统治疗策略，寻找新的治疗突破点。

小剂量持续化疗联合树突状细胞（dendritic cell, DC）的生物免疫治疗是一种全新的治疗模式，除了收获小剂量持续化疗本身抑制肿瘤新生血管的益处外^[3, 4]^，还有望改变肺癌局部的微环境，而此种微环境中产生的细胞因子网络有利于树突状细胞发挥免疫监管的作用，两种方法在肺癌的治疗模式中将会产生互补和叠加的效应。

本试验将利用负荷3LL肺癌细胞的C57/BL6J小鼠，应用多西他赛的小剂量持续化疗联合树突状细胞的生物免疫治疗方法，观察联合治疗的抑瘤效应和肿瘤微环境中与肿瘤新生血管生成相关的细胞因子分泌的变化。

## 1 材料与方法

### 1.1 材料

6周-8周龄的C57/BL6J小鼠为研究对象，雄性，质量20 g左右，SPF级。动物生产许可证SCXK（沪）2007-0001；动物使用许可证SCXK（沪）2008-0043。多西他赛，相对分子质量为862，赛诺菲安万特公司产品；Lewis肺癌细胞（3LL）（中国科学院上海细胞生物研究所）；鼠源性IL-4和GM-CSF（PeprTech）；液态芯片（Luminex）及试剂盒（BD）；微渗析仪及相关导管，探针及连接器。

### 1.2 方法

#### 1.2.1 鼠源性DC的培养

第1天，培养鼠源性骨髓来源的DC：分离小鼠的股关节，取出两条完整的自股关节到踝关节的肢体，针头插入小鼠骨髓腔中，培养液冲洗。在细胞沉淀中依此加入红细胞裂解液和抗体CD4、CD8、CD220以及补体，去除红细胞及T、B淋巴细胞。将DC前体细胞放置于37 ℃培养箱中2 h，收集悬浮细胞，在悬浮细胞中按照GM-CSF 1 U/mL、IL-4 0.5 U/mL加入细胞因子。第6天，收获培养的DC。

#### 1.2.2 不同治疗方式的建立

24只小鼠分别在皮下注射0.5×10^6^个3LL肿瘤细胞；10 d后成瘤。测量小鼠体积，体积计算按照: 1/2长径×短径^2^平衡各组小鼠体积后，随机分4组：对照组（3LL）、DC免疫治疗组（3LL+DC）、小剂量持续化疗组（3LL+chemo）和DC免疫治疗联合小剂量化疗组（3LL+DC+chemo）。四组治疗的具体内容如下：①3LL组：瘤内注射PBS液10 μL；②3LL+DC组：肿瘤种植后11 d、15 d瘤内注射2×10^6^/50 μL DC皮下注入瘤内DC；③3LL+chemo组：每周3次，每次0.2 mg/kg剂量瘤内注射多西他赛；④3LL+DC+chemo组：瘤内11 d、15 d注射2×10^6^/50 μL DC皮下注入瘤内DC，除此之外，每周3次，每次按照0.2 mg/kg剂量瘤内注射多西他赛。绘制小鼠的生存曲线。

#### 1.2.3 微渗析仪技术结合Luminex技术

第22天时，各组小鼠中随机取出一只放置于玻璃笼中，局麻下肿瘤内置入探针，利用微泵将交换液泵入瘤体内，活体状态收集组织间液。小鼠保持清醒，在玻璃笼中正常进食和活动。应用微渗析仪（Microdialysis）联合液态芯片（Luminex）方法分析各组与肿瘤新生血管相关的细胞因子：人碱性成纤维细胞生长因子（basic fibroblast growth factor, bFGF）和血管内皮生长因子（vascular endothelial growth factor, VEGF）。该实验重复3次。

### 1.3 统计学处理

采用SPSS 11.0软件进行统计学分析，*One-way ANOVA*法进行组间比较，生存曲线采用Sigma Plot 11.0软件，*P* < 0.05表示差异具有统计学意义。

## 2 结果

### 2.1 各组荷瘤小鼠的生存曲线

采用不同治疗模式的小鼠生存时间如图 1所示，3LL+DC+chemo能延长荷瘤小鼠的生存时间为（27.6±3.2）天，与3LL组（13.5±2.7）天、3LL+DC组（13.1±2.3）天、3LL+chemo组（11.8±3.0）天相比，差异具有统计学意义（*P*=0.008, *P*=0.01, *P*=0.01）。

**1:**
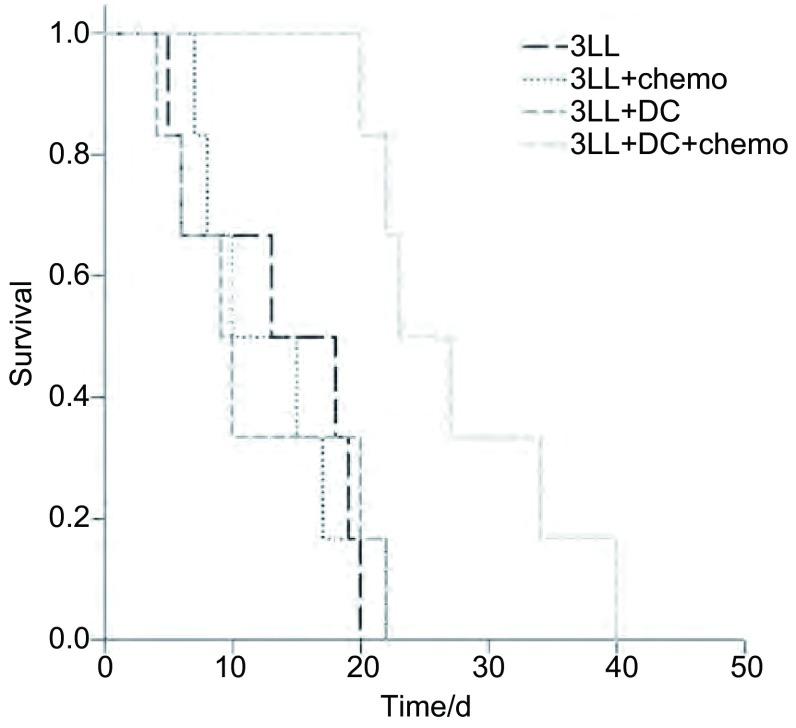
对照组（3LL）、DC免疫治疗组（3LL+DC）、小剂量持续化疗组（3LL+chemo）和DC免疫治疗联合小剂量化疗组（3LL+DC+chemo）的生存曲线。 The survival curve of control group (3LL), dendritic cells (DC) vaccine (3LL+DC), metronomic chemotherapy (3LL+chemo) and metronomic chemotherapy combination with DC vaccine (3LL+DC+chemo).

### 2.2 各组治疗组肿瘤内部细胞因子分泌差异

采用微渗析仪联合Luminex方法检测各组荷瘤小鼠在不同时间分泌的细胞因子：bFGF（表 1）和VEGF（图 2）的表达。结果显示：bFGF的分泌在不同治疗组的不同时段分泌的差异没有统计学意义。而VEGF的分泌在联合治疗组分别在48 h及72 h时有下降趋势，差异具有统计学意义。

**1:** 不同治疗模式下肿瘤微环境中bFGF的分泌 bFGF secretion in tumor microenvironment in different treatments

Group	12 h	24 h	48 h	72 h
3LL	812±51	480±26	669±92	603±301
3LL+DC	596±290	766±107	789±200	740±165
3LL+chemo	455±109	576±105	696±45	962±111
3LL+DC+chemo	687±209	892±363	789±211	904±121
bFGF, basic fibroblast growth factor. There was no significant difference in bFGF secretion in different groups.				

**2:**
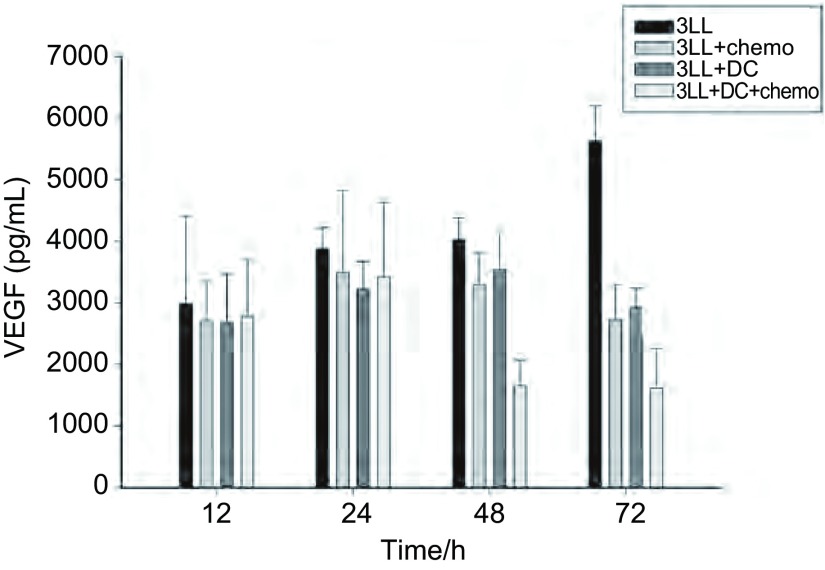
不同治疗模式下肿瘤微环境中血管内皮生长因子（vascular endothelial growth factor, VEGF）的分泌。3LL瘤体内VEGF的分泌随着肿瘤的生长呈升高趋势。各治疗组内的VEGF分泌较3LL组都有下降的趋势。在3LL+DC+chemo组，这种抑制作用在48 h、72 h时最为明显，与各组之间的差异具有统计学意义（*P* < 0.05）。 VEGF secretion in tumor microenvironment in different treatments. With the growth of tumor, the secretion of VEGF in 3LL tumor increased. Compared with 3LL group, VEGF secretion was reduced in the other three groups. This inhibition presented obviously in 48 h and 72 h in metronomic chemotherapy combination with DC vaccine group, compared with other groups, *P* < 0.05.

## 3 讨论

2004年Kerbel等^[3]^在Nature Rev Cancer杂志上报道，小剂量持续化疗较之于传统化疗的优势是，在杀伤癌细胞的同时，还能抑制癌周微环境中新生血管内皮细胞的生长。这种小剂量持续化疗以投予频繁的化疗药物与短暂休息间隔交替进行为特点。其应用于临床最大的益处将是患者对此接受度高并且有很好的耐受性。多西他赛是一种新颖的细胞毒药物，其作用机制是加强微管蛋白聚合作用和抑制微管解聚作用，导致稳定的非功能性微管束形成，因而能破坏肿瘤细胞的有丝分裂。目前，多西他赛已经成为肺癌患者化疗的首选药物。然而，在常规剂量下，经常会出现强烈的副反应，包括胃肠道、血液系统的毒副反应，严重者甚至危及患者生命。因此，应用小剂量持续疗法，既能抑制肿瘤新生血管，又能起到减轻化疗毒副反应的作用是一种新颖的化疗方法。

DC是唯一能向初始T细胞递呈抗原，并激发T细胞及B细胞免疫应答的、功能最强的专职性抗原递呈细胞，在调节机体免疫反应，尤其在启动抗肿瘤免疫反应中起关键作用。DC激发免疫反应有如下特点：①极少量的DC就能激发强大的T细胞免疫应答；②可以激活静止状态的T细胞群；③在体内可以同时致敏CD4^+^T辅助细胞和CD8^+^T杀伤细胞^[5, 6]^。但目前单纯应用DC免疫治疗肺癌尚未获得预期的疗效，究其原因，可能是以DC为基础的免疫生物疗法的有效性更多地体现在改变肿瘤局部的微环境，防治肿瘤转移，从而提高荷瘤宿主的带瘤生存期，而不是直接的杀瘤效应^[7, 8]^。因此，我们设想，如能以多西他赛小剂量持续化疗法联合DC免疫生物疗法，可望获得更强大的抗肺癌效果。在此试验中，我们应用荷瘤动物验证我们的想法。

VEGF是在促进肿瘤血管生成中起主要作用的细胞因子，促肿瘤血管生成的其他因子如人碱性成纤维细胞生长因子（basic fibroblast growth factor, bFGF）、血小板源性生长因子（platelet derived growth factor, PDGF）、肿瘤坏死因子*α*（tumor nectosis factor-*α*, TNF-*α*）、表皮生长因子（epidermal growth factor, EGF）等，这些生长因子其作用主要通过诱导VEGF表达实现促进肿瘤生长、浸润和转移的作用。VEGF及其受体信号通路激活可促进内皮细胞存活、增殖与迁移，也可促进内皮祖细胞被招募至血管生成活跃部位。肿瘤血管生成是一个非常复杂的过程，VEGF、bFGF对血管生成的多个环节，如血管内皮细胞的增殖、分化、迁移和管腔状结构的形成、血管内皮基底膜的降解等均有明显的促进作用。VEGF及其受体的表达还受到bFGF的调控，bFGF在体内外均能诱导VEGF的合成，还能上调VEGF受体Flk-l的表达。

我们的试验结果显示：小剂量持续化疗联合树突状细胞生物免疫治疗能够延长荷瘤小鼠的生存，一定程度上在于联合治疗抑制瘤内VEGF的分泌，可能通过瘤体内VEGF的抑制产生了抑制肿瘤新生血管的形成。除此之外，小剂量持续化疗联合树突状细胞生物免疫治疗比之于单一的治疗方式获得生存获益的原因在于化疗本身刺激了肿瘤抗原的广泛释放，刺激了免疫细胞发挥强大的抗原提呈作用。更多的内在机制需要大样本的实验研究证实。
